# miR-153-3p Targets βII Spectrin to Regulate Formaldehyde-Induced Cardiomyocyte Apoptosis

**DOI:** 10.3389/fcvm.2021.764831

**Published:** 2021-12-15

**Authors:** Panyu Yang, Yanyan Yang, Xiangqin He, Pin Sun, Ying Zhang, Xiaoxia Song, Yu Tian, Tingyu Zong, Jianmin Ma, Xiaofei Chen, Qifeng Lv, Tao Yu, Zhirong Jiang

**Affiliations:** ^1^Department of Cardiac Ultrasound, The Affiliated Hospital of Qingdao University, Qingdao, China; ^2^Department of Immunology, Basic Medicine School, Qingdao University, Qingdao, China; ^3^Department of Regenerative Medicine, The Affiliated Hospital of Qingdao University, Qingdao, China

**Keywords:** congenital heart disease, formaldehyde, microRNA, cardiomyocyte, apoptosis

## Abstract

**Background:** Formaldehyde (FA) is ubiquitous in the environment and can be transferred to the fetus through placental circulation, causing miscarriage and congenital heart disease (CHD). Studies have shown that βII spectrin is necessary for cardiomyocyte survival and differentiation, and its loss leads to heart development defects and cardiomyocyte apoptosis. Additionally, previous studies have demonstrated that miRNA is essential in heart development and remodeling. However, whether miRNA regulates FA-induced CHD and cardiomyocyte apoptosis remains unclear.

**Methods:** Using commercially available rat embryonic cardiomyocytes and a rat model of fetal cardiomyocyte apoptosis. Real-time quantitative PCR (RT-qPCR) and Western blot were performed to examine the level of miR-153-3p, βII spectrin, caspase 7, cleaved caspase7, Bax, Bcl-2 expression in embryonic cardiomyocytes and a rat model of fetal cardiomyocyte apoptosis. Apoptotic cell populations were evaluated by flow cytometry and Tunel. Luciferase activity assay and RNA pull-down assay were used to detect the interaction between miR-153-3p and βII spectrin. Masson's trichrome staining detects the degree of tissue fibrosis. Fluorescence *in situ* hybridization (FISH) and Immunohistochemistry were used to detect the expression of miR-153-3p and βII spectrin in tissues.

**Results:** Using commercially available rat embryonic cardiomyocytes and a rat model of fetal cardiomyocyte apoptosis, our studies indicate that miR-153-3p plays a regulatory role by directly targeting βII spectrin to promote cardiomyocyte apoptosis. miR-153-3p mainly regulates cardiomyocyte apoptosis by regulating the expression of caspase7, further elucidating the importance of apoptosis in heart development. Finally, the results with our animal model revealed that targeting the miR-153-3p/βII spectrin pathway effectively regulated FA-induced damage during heart development. Recovery experiments with miR-153-3p antagomir resulted in the reversal of FA-induced cardiomyocyte apoptosis and fetal cardiac fibrosis.

**Conclusion:** This study investigated the molecular mechanism underpinning the role of βII spectrin in FA-induced CHD and the associated upstream miRNA pathway. The study findings suggest that miR-153-3p may provide a potential target for the clinical diagnosis and treatment of CHD.

## Introduction

Congenital heart disease (CHD) affects approximately 1% of newborns every year and is a common birth defect (1). The etiology of CHD involves a variety of genetic and environmental factors, among which an estimated 400 genes are associated with its pathogenesis (1, 2). Gene mutations that encode transcription factors, chromatin modifiers, and cell signal transducers can interfere with essential cell type specification, differentiation, and mode for heart development, thereby causing disturbances in cardiac structure and function (2). About 80% of CHD is caused by various combinations of genetic and environmental factors (3). The main cause of death in CHD patients is arrhythmia, followed by congestive heart failure; however, myocardial infarction has become the leading cause of death in the past ten years (4). Interestingly, CHD is closely related to cardiomyocyte apoptosis (5–7). As many CHD patients suffer from complications later in life, among which heart failure and arrhythmia are the most prominent, patients often undergo repeated operations at enormous costs. Therefore, studying the pathogenesis of CHD has important practical significance for its prevention and treatment, including improving the birth quality of the population by reducing birth defects.

Formaldehyde (FA) is an environmental and occupational pollutant that is widely present in people's lives. Studies have demonstrated that FA is genetically toxic in a variety of *in vitro* models, as well as in humans and experimental animals (8, 9). FA reportedly exerts toxic effects on adult male rat reproductive health, the functional mechanism of which may be due to cell apoptosis (10). Specifically, FA exposure has an adverse effect on semen quality (11). Moreover, a survey investigating the influence of fathers' occupational exposure to FA revealed that when fathers are exposed to FA, the risk of prolonged time to pregnancy and spontaneous abortion is significantly increased (12). Ovarian toxicity due to FA is also dose-dependent, affecting the ovaries by inducing oxidative stress (13). Additional studies have confirmed that exposure to FA during pregnancy may increase the risk of spontaneous abortion (14). Further, a correlation was reported between FA exposure and decreased biparietal diameter in the second trimester (15). Therefore, FA exposure is closely related to reproductive toxicity. A sufficiently high dose of FA induced oxidative stress and cardiomyocyte apoptosis in pregnant rats and offspring, which was reversed with vitamin E supplementation (16). However, present studies investigating the mechanism of FA pollution exposure on embryonic heart development remain incomplete, and the mechanism warrants further exploration.

βII spectrin is a cytoskeletal protein that exists in all nucleated cells. Studies have shown that βII spectrin is necessary for the healthy development of various organs, including nerve, liver, and heart (17). The function of βII spectrin includes establishing and maintaining cell structure. In addition, regulating various cell functions, such as apoptosis, cell adhesion, and cell cycle regulation. It is worth noting that βII spectrin dysfunction is related to embryonic lethality (18). Recently detected changes in βII spectrin expression in tumors demonstrated that it may be associated with the occurrence and development of cancer. Indeed, βII spectrin mutations and disorders are related to various developmental disorders and diseases. However, the potential role of βII spectrin in embryonic heart development remains unclear.

Non-coding RNA (ncRNA) refers to RNA that is transcribed from the genome but performs its biological function at the RNA level without being translated into protein. Wang et al. (19) reported that lncRNA-CARL (cardiac apoptosis-related) inhibits hypoxia-induced mitochondrial division and cardiomyocyte apoptosis by weakening the function of miR-539 and downregulating the expression of prohibitin 2 (PHB2). They also found that circular RNA (circRNA) can be mediated by upregulating the expression of miRNA-dependent mitochondrial 18 KDa protein (MTP18), leading to cardiomyocyte death (20). Another study found that lncRNA-CPR (cardiomyocyte proliferation regulator) is critical in regulating cardiomyocyte proliferation and cardiac repair (21). The discovery of ncRNA has provided new insights into the mechanism of cardiovascular disease (22–27). miRNA reportedly plays a vital role in heart development and remodeling (28), and is essential in post-transcriptional regulation. Studying the roles of miRNA in cardiac development and disease will greatly improve our basic knowledge of the molecular mechanisms underlying heart development, and is essential for the development of new diagnostic markers and treatment strategies for CHD.

This study investigated the differential expression of miR-153-3p in FA treated embryonic cardiomyocytes and in an animal model of fetal cardiomyocyte apoptosis, providing a theoretical basis for screening molecular targets that regulate cardiomyocyte function, and also presenting a potential new target for the diagnosis and treatment of CHD.

## Materials and Methods

### Animal Experiment

Twenty female and ten male Sprague-Dawley rats (250–300 g) were raised at the Animal Center of the Qingdao University Medical College. The rats received regular feeding and were exposed to natural light and a room temperature of 19–24°C. After acclimation for a few days, the rats were caged according to a 2:1 ratio of males to females. Vaginal monitoring was performed the next morning; vaginal plug formation was considered day 1 of pregnancy. Pregnant rats were randomly divided into the following groups (*n* = 5): low FA exposure (0.2 mg/kg), medium FA exposure (2 mg/kg), high FA exposure (20 mg/kg) (29), and control group (normal saline). On the 7th day of pregnancy, the appropriate concentration of FA (or saline) was injected into the abdominal cavity once daily for 12 days. The pregnant rats were closely monitored and anesthetized via intraperitoneal injection of chloral hydrate (0.3 mL/100 g) on the 12th day of treatment exposure.

The pregnant rats were randomly divided into three groups (*n* = 5, *in vivo* recovery experiment): control, medium FA concentration, and medium FA concentration together with miR-153-3p antagomir. Animal breeding conditions are as described above. From the first day to the fourth day of FA treatment, miR-153-3p antagomir (2 mg/kg) was injected into the rat tail vein every day. The pregnant rats were closely monitored and anesthetized via intraperitoneal injection of chloral hydrate (0.3 mL/100 g) on the 12th day of FA exposure. Fetal rat hearts were then collected, and tissue samples were either paraffin-sectioned and stained or homogenized for total RNA and total protein extraction. The Research Ethics Committees of the Affiliated Hospital of Qingdao University approved this study, and all experiments were conducted following the principles of the Declaration of Helsinki.

### Cell Culture, Transfection, and Treatment

Rat embryonic cardiomyocyte cell line H9C2 (American Type Culture Collection, Manassas, VA, USA) was routinely resuscitated using DMEM complete medium (Gibco, Grand Island, NY, USA) containing 10% fetal bovine serum (FBS) (ExCell Bio, Shanghai, China). Cells were cultured in a constant temperature incubator at 37°C under 5% CO_2_ until reaching 95% confluency. Cells were transfected with Lipofectamine 3,000 (Invitrogen, Carlsbad, CA, USA) according to the manufacturer's instructions. miR-153-3p mimic (GenePharma, Shanghai, China): 5′-UUGCAUAGUCACAAAAGUGAUC−3′, 5′-UCACUUUUGUGACUAUGCAAUU-3′; mimic NC (GenePharma): 5′-UUCUCCGAACGUGUCACGUTT-3′, 5′-ACGUGACACGUUCGGAGAATT-3′. miR-153-3p inhibitor (GenePharma): 5′-GAUCACUUUUGUGACUAUGCAA−3′; inhibitor NC (GenePharma): 5′-CAGUACUUUUGUGUAGUACAA−3′. Si-βII spectrin: 5′-GGACAUGUCUUAUGAUGAATT-3′, 5′-UUCAUCAUAAGACAUGUCCTT-3′; siRNA control: 5′-UUCUCCGAACGUGUCACGUTT-3′, 5′-ACGUGACACGUUCG GAGAATT-3′ (GenePharma). For the FA induction experiment, H9C2 cells were treated with 0, 50, 100, and 150 μmol/L FA (Sigma-Aldrich, St. Louis, MO, USA) for different times (0, 1, 3, 6, 12, and 24 h).

### RNA Isolation and Quantitative Real-Time PCR

TRIzol reagent (Invitrogen) was used to extract RNA from H9C2 cells and fetal heart tissues. Additionally, tissue samples were cut, homogenized, and mixed with chloroform prior to RNA isolation. The precipitate was thoroughly washed with 75% ethanol prepared with DEPC-treated water and finally dried to obtain isolated RNA. After measuring the concentration, reverse transcription of RNA into cDNA is done by PrimeScript Reverse Transcription (RT) reagent kit (Takara Bio, Kyoto, Japan). Hieff UNICON Power qPCR SYBR Green Master Mix (Yeasen Biotechnology Co., Ltd., Shanghai, China) was used for RT-qPCR (Bio-Rad, American), with GAPDH used as the internal reference. All experimental steps were performed in accordance with the manufacturer's instructions. Amplification conditions: denaturation: 95°C, 5 min; annealing: 60°C, 30 s; extension: 95°C, 5 s; 40 cycles. The PCR primers are shown in Table 1.

**Table 1:** Primer sequences for RT-qPCR.

**Primer**	**Sequences (5**′** → 3**′**)**
GAPDH F	GTAACGAATTCGACGT
GAPDH R	CAAGCTAACTTGCGAAACGT
U6 F	CTCGCTTCGGCAGCACA
U6 R	TGGTGTCGTGGAGTCG
miR-153-3p	TTGCATAGTCACAAAAGTGATC
βII spectrin F	GGCCAGACTCTGTTATAGCTTGG
βII spectrin R	CCCTGAATGGTTTTACTCCACTGC
miR-28-3p	CACTAGATTGTGAGCTCCTGGA
miR-323-3p	CACATTACACGGTCGACCTCT
Bax F	CATGAAGACAGGGGCCTTTTTG
Bax R	TCAGCTTCTTGGTGGATGCGTC
Bcl 2 F	GGGCTACGAGTGGGATACTGGAG
Bcl 2 R	CGGGCGTTCGGTTGCTCT
Caspase 7 F	TATCAACGACACCGACGCTAAT
Caspase 7 R	GCCTGGAACCGTGGAGTAAG
PCNA F	CATATTGGAGATGTGGTGTGGTGAT
PCNA R	CATACTGAGTGTTACTGTAGGAGAC
α-SMA F	CTGTTATAGGTGGTTTCGTGGA
α-SMA R	AGAGCTACGAACTGCCTGA
NKX 2.5 F	GCCAACAGCAACTTCGTGA
NKX 2.5 R	TCCCTACCAGGCTCGGAT
Dystrophin F	CAAGTGGCAAGTTCAACCG
Dystrophin R	CAGACGCATCCAGTCAAGG

### Western Blot Analysis

Cells were lysed with an appropriate amount of RIPA lysis buffer (Solarbio, Beijing, China) for 10 min on ice. The mixture was then centrifuged, and bicinchoninic acid (BCA; Solarbio) method determines protein concentration, employing a microplate reader to detect the absorbance of the protein sample at 562 nm. Proteins were separated by 7–12.5% SDS-PAGE (Solarbio), and transferred to polyvinylidene fluoride (PVDF) membrane. The PVDF membrane was blocked with 5% skim milk. Then, it incubated with primary antibody (βII spectrin, Abcam, ab72239; Cleaved caspase 7, Cell Signaling Technology, #8438S; Caspase 7, Abclonal, A1524; Bax, Abclonal, A19684; Bcl 2, Zenbio, 250414), followed by secondary antibody (Abcam, Cambridge, UK). Proteins were detected using the Fusion Solo S system (Vilber, Paris, France).

### Immunohistochemistry

Fetal heart tissue was fixed in 4% FA solution and paraffin-sectioned. Sections were then deparaffinized in xylene, rinsed with PBS, and incubated in 3% H_2_O_2_ in 50% methanol at 37°C for 30 min. After eliminating endogenous peroxidase activity, and incubated in a protein blocking solution (Bio-Genex, San Ramon, CA, USA) for 30 min. Sections were incubated with βII spectrin antibody (1:100; ab72239, Abcam) overnight at 4°C to block non-specific binding. Then, goat anti-rabbit secondary antibody for 30 min. The peroxidase substrate diaminobenzidine was used for the reaction. Hematoxylin counterstain, observed under a microscope (Nikon, Tokyo, Japan).

### Fluorescence *in situ* Hybridization

Fetal heart tissue was embedded, frozen, sectioned, and FISH was performed to detect the expression of miR-153-3p, as previously described (30). The sequence of the miR-153-3p probe (GenePharma) used for FISH was 5′-CY5-GATCACTTTTGTGACTATGCAA-3′; NC probe: 5'-CY5-TTAGAGGCATCTCGTA ATCTAT-3′. Nuclei were stained using DAPI and slides were mounted using Vectashield mounting agent (Vector Labs, Burlingame, CA, USA).

### RNA Pull-Down Assay

H9C2 cells were seeded into a 10 cm dish. The cells were subsequently scraped off and a glass grinder was used to fully lyse the cells on ice. Centrifuge at low temperature and collect the supernatant for further use. The miR-153-3p probe (GenePharma) sequences were: miR-153-3p WT 5′-bio- UUGCAUAGUCACA AAAGUGAUC-3′, miR-153-3p Mut 5′-bio- AACGUAUGUCACAAAAGUGAUC-3′. The scrambled control probe was: 5′-bio- AUAAGUACUGUAGUAGAACUCC-3′. The probe was dissolved according to the manufacturer's protocol, and 10 μL of the dissolved probe solution was mixed with 30 μL Pierce Streptavidin Agarose (Thermo-Fisher Scientific, Paisley, UK) and treated with buffer (31). Finally, the lysed sample was mixed with the Pierce Streptavidin Agarose-probe solution, and incubated overnight at 4°C. TRIzol reagent was added to extract RNA, which was then used for βII spectrin detection by RT-qPCR.

### Luciferase Activity Assay

The reconstructed pmirGLO luciferase vector (GenScript Biotech, Piscataway, NJ, USA) contained the 3′UTR fragment of wild-type βII spectrin (βII spectrin-Wt) with a miR-153-3p binding site or that of mutant βII spectrin without a miR-153-3p binding site (βII spectrin-Mut). To measure luciferase activity, 293T cells (Shanghai Institute of Biochemistry and Cell Biology, Shanghai, China) were seeded into a 24-well plate. Use Lipofectamine 3,000 reagent to co-transfect with reconstituted luciferase vector and miR-153-3p mimic or NC mimic. After 48 h of transfection, use Dual-Luciferase Reporter Gene Assay kit (Meilunbio, Dalian, China) to detect luciferase activity according to the manufacturer's instructions.

### TUNEL Staining

H9C2 cells were seeded into a 24-well plate and cardiomyocyte apoptosis was evaluated under different conditions using TUNEL assay, performed using the TUNEL Apoptosis Detection kit (Yeasen Biotechnology Co., Ltd.) according to the manufacturer's instructions. Cells in each well were incubated with 50 μL TUNEL mixture at 37°C for 1 h. Stain cell nuclei using DAPI. The apoptotic rate was determined as the number of apoptotic cells (red)/total number of cells (blue) × 100%. Cells were observed under a fluorescence microscope (Nikon, Tokyo, Japan). The optical density was measured using ImageJ v1.5.1 software.

### CCK-8

Cell viability was measured using Cell Counting Kit-8 (Yeasen Biotechnology Co., Ltd.). A total of 4 × 10^3^ H9C2 cells per well were seeded into a 96-well plate. According to the manufacturer's instructions, 10 μL CCK-8 solution was added to 100 μL medium. Reaction conditions: 37 °C, 2 h. The absorbance (Bio-tek, American) was measured at 450 nm.

### Flow Cytometry Analysis

Apoptosis was evaluated using the Annexin V-FITC Cell Apoptosis Detection kit (Meilunbio). H9C2 cells were subsequently collected, treated with trypsin without EDTA, washed with PBS, and finally centrifuged. According to the manufacturer's instructions, the cell pellet was resuspended in binding buffer and incubated with annexin V-FITC reagent and propidium iodide in the dark. Finally, the cells were analyzed using a Beckman FC 400 MPL flow cytometer (Beckman Coulter Inc., Brea, CA, USA).

### Masson's Trichrome Staining

Fetal heart tissue was fixed with 10% FA at room temperature for 24 h, then decalcified, dehydrated, permeated with xylene, embedded in wax. Wiegert's iron hematoxylin solution (Sigma-Aldrich) was used to stain the nuclei for 5 min. First stain with 0.7% Masson's Trichrome Stain solution (Sigma-Aldrich) for 10 min after rinsing with distilled water. Rinsing: 2% glacial acetic acid; differentiation: in phosphomolybdic acid, 4 min. Continue dyeing with 2% aniline blue dye solution (Sigma-Aldrich). After dehydration, dewaxing, and fixation with neutral resin, the image was taken with an optical microscope (Nikon).

### Statistical Analysis

Statistical analysis was performed using GraphPad Prism version 8.0 (GraphPad Software, La Jolla, CA, USA) and IBM SPSS Statistics version 25.0 (IBM Corp., Armonk, NY, USA) software. The results are expressed as mean ± standard deviation values. Statistical differences were determined by unpaired Student's *t*-test, one-way analysis of variance (ANOVA), and Tukey's *post- hoc* test or Mann-Whitney test, depending on the distribution of variables. A *p*-value < 0.05 was considered statistically significant. Each experiment was repeated at least three times.

## Results

### The Effect of Formaldehyde on βII Spectrin and Apoptosis *in vivo* and *in vitro*

**Figure 1:**
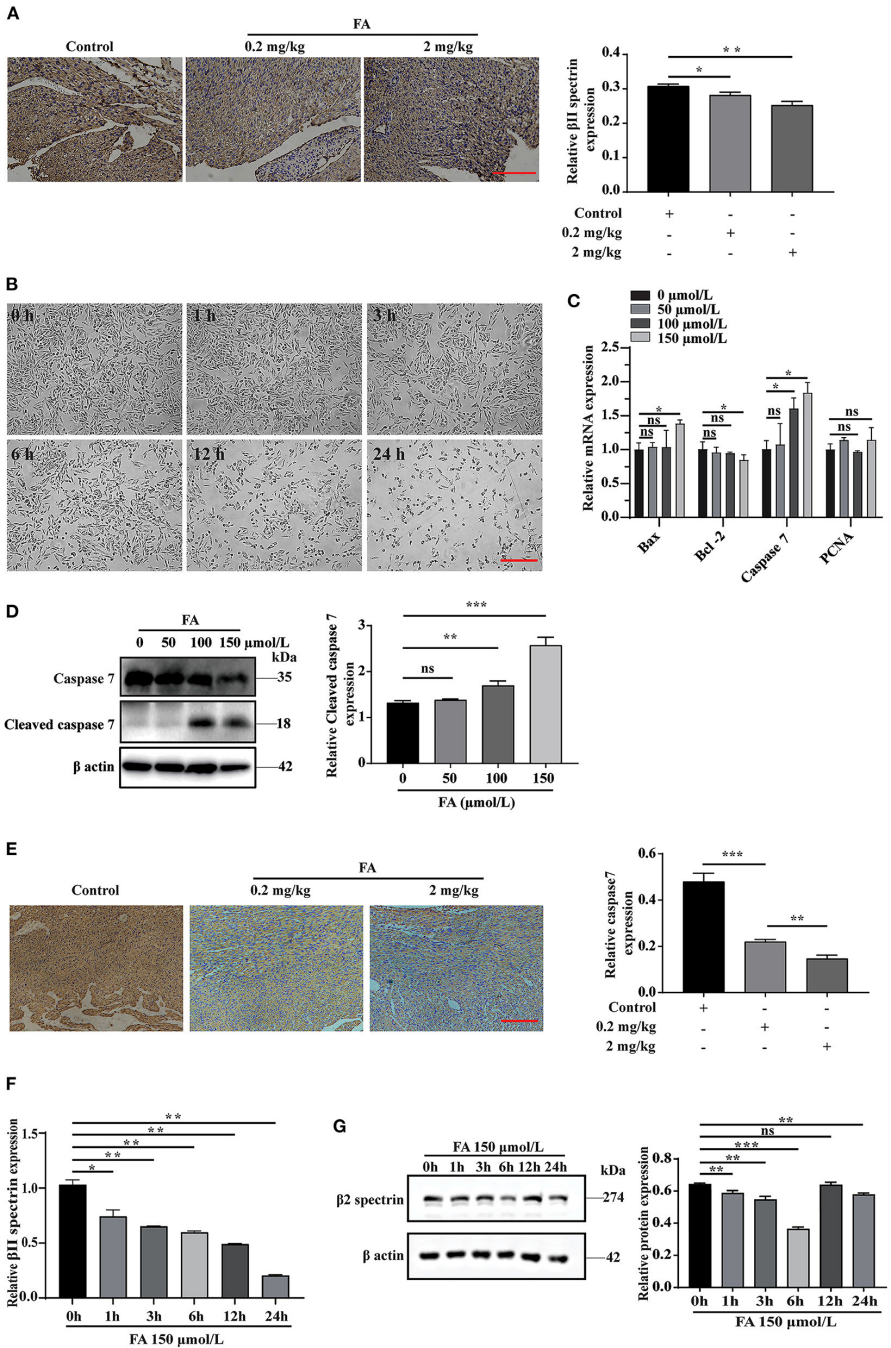
The effect of formaldehyde on βII spectrin and apoptosis *in vivo* and *in vitro*. **(A)** Immunohistochemistry was used to detect the expression of βII spectrin in the heart of fetal rats after intraperitoneal injection of formaldehyde. **(B)** Treat H9C2 cells with formaldehyde at a concentration of 150 μmol/L, and observe the growth status of the cells. **(C)** RT-qPCR was used to detect the expression of apoptosis and proliferation related proteins Caspase7, Bax, Bcl-2, PCNA under different formaldehyde concentrations. **(D)** WB detects the expression of Cleaved caspase7 and Caspase7 at a concentration of 150 μmol/L. **(E)** Immunohistochemistry was used to detect the expression of caspase7 in the heart of fetal rats after intraperitoneal injection of formaldehyde. **(F)** RT-qPCR detects the expression of βII spectrin at a concentration of 150 μmol/L formaldehyde at different times. **(G)** WB detects the expression of βII spectrin at 150 μmol/L formaldehyde concentration at different time treatments. Results were quantified by ImageJ software. Results were quantified by ImageJ software. Data are presented as mean ± SD. Scale bars, 200 μm. *n* = 3. *p < 0.05 vs. Ctl, **p < 0.01 vs. Ctl; ***p < 0.001 vs. Ctl.

The role of βII spectrin in embryogenesis, especially in heart development, has been partially determined in previous studies. Specifically, βII spectrin is essential for the survival and differentiation of cardiomyocytes, and its loss can lead to defects in cardiac development and the inability to thicken the ventricular wall (32). In addition, FA is closely associated with apoptosis (33, 34). At present, whether βII spectrin is involved in the molecular mechanism underlying FA-induced fetal heart development defects remains unclear. Immunohistochemical analysis revealed that βII spectrin expression was reduced in fetal rat hearts after intraperitoneal injection of FA (Figure 1A). According to previous studies (35, 36), FA can induce apoptosis in lung cells and mouse bone marrow cells. In our study, with the extension of the treatment time, the concentration of 150 μmol/L FA also significantly reduced the number of H9C2 cells (Figure 1B). RT-qPCR analysis revealed that FA did not significantly affect the expression of proliferation-related protein PCNA (proliferating cell nuclear antigen) (Figure 1C). Furthermore, we found that at a concentration of 150 μmol/L FA, the expression of apoptosis-related proteins Bax and caspase7 increased in H9C2 cells, while that of apoptosis-related Bcl-2 decreased (Figure 1C). Western blotting revealed that the expression of cleaved caspase7 increased significantly when cells were treated with 150 μmol/L FA, whereas the expression of caspase7 decreased significantly (Figure 1D). Immunohistochemical analysis revealed that caspase7 expression was reduced in fetal rat hearts after intraperitoneal injection of FA (Figure 1E). Further RT-qPCR analysis indicated that βII spectrin expression gradually decreased with time when cells were treated with 150 μmol/L FA, which was confirmed by western blotting (Figures 1F,G). Based on these results, we speculate that certain FA concentrations can lead to increased cardiomyocyte apoptosis, which might lead to the onset and progression of CHD.

### miR-153-3p Targets Regulation of βII Spectrin

**Figure 2:**
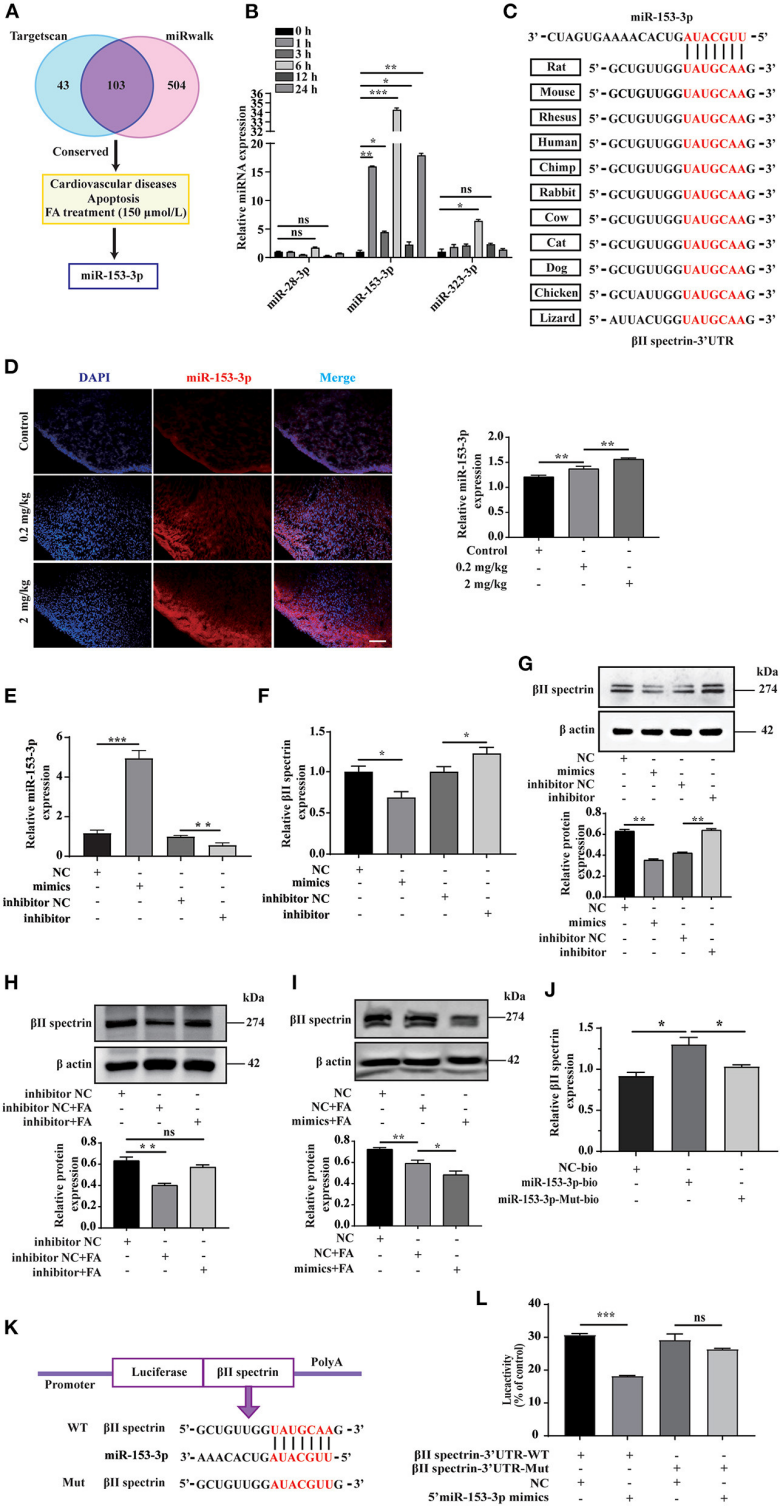
miR-153-3p targets regulation of βII spectrin. **(A)** Using Targetscan and miRwalk, we initially screened conserved miRNAs, then selected miRNAs known to be related to cardiovascular disease and apoptosis, and finally selected miRNAs that changed most significantly under 150 μmol/L formaldehyde treatment. **(B)** 150 μmol/L formaldehyde was used to treat H9C2 cells at different times, and it was found that miR-153-3p increased the most. **(C)** The results of conservative analysis show that miR-153-3p is highly conservative. **(D)** Fish experiment to detect the expression of miR-153-3p in the heart tissue of fetal rats treated with different formaldehyde concentrations. **(E)** RT-qPCR detects the transfection efficiency of miR-153-3p mimics and inhibitor transfected into H9C2 cells. **(F)** RT-qPCR detection of βII spectrin expression in H9C2 cells after transfection of miR-153-3p mimics and inhibitor. **(G)** WB detection of βII spectrin expression in H9C2 cells after transfection of miR-153-3p mimics and inhibitor. **(H)** WB detects the interaction between miR-153-3p inhibitor and βII spectrin under pathological stimuli. **(I)** WB detects the interaction between miR-153-3p mimics and βII spectrin under pathological stimuli. **(J)** RNA pulldown experiment detects that the miR-153-3p biotin probe pulls βII spectrin significantly at the mRNA level compared with the control and mutant probes. **(K)** βII spectrin 3'UTR (wt 3'UTR and mut 3'UTR) luciferase reporter gene insertion pattern. **(L)** The luciferase reporter gene detects the direct binding of βII spectrin and miR-153-3p. Results were quantified by ImageJ software. Data are presented as mean ± SD. Scale bars, 200 μm. *n* = 3. *p < 0.05 vs. Ctl, **p < 0.01 vs. Ctl; ***p < 0.001 vs. Ctl.

We employed bioinformatics analysis to predict the upstream miRNA of βII spectrin in rats using the TargetScan and miRWalk databases to identify 103 miRNAs, shown in the Venn diagram. We screened conserved miRNAs reportedly related to cardiovascular disease and apoptosis in the literature, and those displaying significant changes in expression when cells were treated with 150 μmol/L FA (Figure 2A). Among them, the expression of miR-153-3p increased most significantly (Figure 2B). Notably, miR-153-3p is highly conserved in humans, rats, and mice (Figure 2C). In order to further verify whether the *in vitro* miR-153-3p results were consistent in the animal model, FISH was used to detect the expression of miR-153-3p in heart tissues of fetal rats treated with different FA concentrations (0.2 mg/kg and 2 mg/kg). Consistently, miR-153-3p expression was increased in the FA-treated groups, with the greatest increase observed in the 2 mg/kg treatment group (Figure 2D). We speculate that miR-153-3p is essential in the regulatory pathway responsible for FA-induced reduction of βII spectrin expression.

To further verify the interaction between miR-153-3p and βII spectrin *in vitro*, we transfected H9C2 cells with the miR-153-3p mimic or inhibitor with high transfection efficiency (Figure 2E). RT-qPCR was employed to detect changes in βII spectrin expression in H9C2 cells after transfection, indicating that βII spectrin expression in the miR-153-3p mimic-transfected group was lower than that in the control group. However, βII spectrin expression in the miR-153-3p inhibitor-transfected group was increased (Figure 2F). We also verified these results at the protein level by WB (Figure 2G). Moreover, the interaction between miR-153-3p and βII spectrin was further verified under pathological stimulus conditions, revealing that transfection with the miR-153-3p inhibitor significantly restored the FA-induced decrease in βII spectrin expression (Figure 2H). Consistently, βII spectrin expression was reduced in cells treated with the miR-153-3p mimic and FA (Figure 2I). We speculated that miR-153-3p directly interacts with βII spectrin. Therefore, the RNA pull-down assay was performed using the miR-153-3p biotin probe, indicating that βII spectrin significantly bound to miR-153-3p at the mRNA level, compared with the control group (Figure 2J). In order to further verify the possibility of direct binding between βII spectrin and miR-153-3p, we co-transfected 293T cells with the reconstructed luciferase vectors (with βII spectrin-Wt and βII spectrin-Mut 3'UTR) and the miR-153-3p mimic (Figure 2K). The luciferase activity of 293T cells co-transfected with βII spectrin-Wt 3'UTR and the miR-153-3p mimic was significantly lower than that of cells co-transfected with βII spectrin-Mut 3'UTR and the miR-153-3p mimic (Figure 2L).

### Overexpression of miR-153-3p Promotes Apoptosis of H9C2 Cells

**Figure 3:**
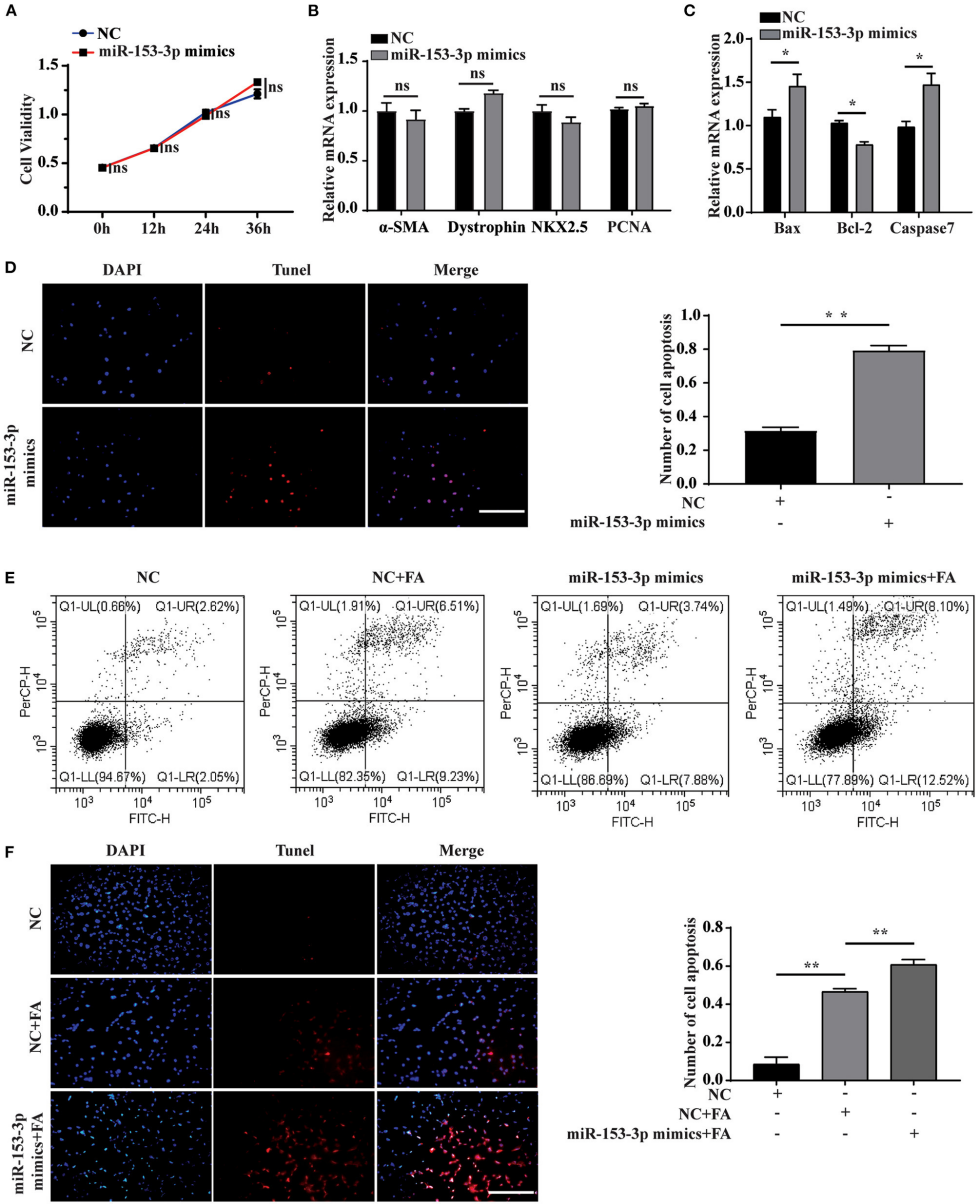
Overexpression of miR-153-3p promotes apoptosis of H9C2 cells. **(A)** CCK8 detects the proliferation of H9C2 cells 0, 12, 24, and 36 h after miR-153-3p mimics are transfected. **(B)** RT-qPCR was used to detect the expression of myocardial differentiation markers α-SMA, Dystrophin and NKX2.5 and the proliferation marker PCNA after miR-153-3p mimics were transfected into H9C2 cells. **(C)** RT-qPCR detection of the expression of Bax, Bcl-2 and Caspase7 after miR-153-3p mimics were transfected into H9C2 cells. **(D)** Tunel experiment to detect the apoptosis of miR-153-3p mimics after transfection 24 h. **(E)** Cell flow cytometry tests to detect cell apoptosis with 150 μmol/L formaldehyde after transfection of miR-153-3p mimics. **(F)** In the Tunel experiment, after transfection of miR-153-3p mimics, 150 μmol/L formaldehyde was continued to stimulate cell apoptosis. Results were quantified by ImageJ software. Data are presented as mean ± SD. Scale bars, 200 μm. *n* = 3. *p < 0.05 vs. Ctl, **p < 0.01 vs. Ctl; ***p < 0.001 vs. Ctl.

We explored the effect of miR-153-3p overexpression on cardiomyocyte function by transfecting H9C2 cells with the miR-153-3p mimic and examining cell behavior at 0, 12, 24, and 36 h. The CCK-8 assay revealed that cell proliferation was not significantly affected by transfection with the miR-153-3p mimic (Figure 3A). Previous research has shown that α-SMA, dystrophin, and NKX2.5 are cardiomyocyte differentiation markers, and PCNA is associated with proliferation. Therefore, we transfected H9C2 cells with the miR-153-3p mimic for 24 h and assessed the expression of the above-mentioned markers. RT-qPCR analysis indicated that expression changes in the differentiation and proliferation markers were not significant (Figure 3B). Bax, Bcl-2, and Caspase7 have been previously associated with apoptosis in liver cancer, and βII spectrin expression is closely related to that of caspase7. Therefore, we measured the expression of Bax, Bcl-2, and caspase7 after transfecting H9C2 cells with the miR-153-3p mimic for 24 h. The results demonstrated that Bax and caspase7 expression increased significantly, while that of Bcl-2 decreased significantly (Figure 3C). The TUNEL assay results confirmed that transfection with the miR-153-3p mimic for 24 h significantly increased the apoptosis of H9C2 cells compared with the control group (Figure 3D). We then stimulated the transfected cells with 150 μmol/L FA for 24 h and performed cell flow cytometry experiments. Consistently, apoptosis rate was strongly activated after transfection of miR-153-3p mimics (11.62%) than NC group (4.67%) as well as FA induced condition (Figure 3E). The TUNEL assay results confirmed these findings (Figure 3F). Taken together, the results demonstrate that overexpression of miR-153-3p promoted the apoptosis of H9C2 cells.

### Knockdown of miR-153-3p Inhibits Apoptosis of H9C2 Cells

**Figure 4:**
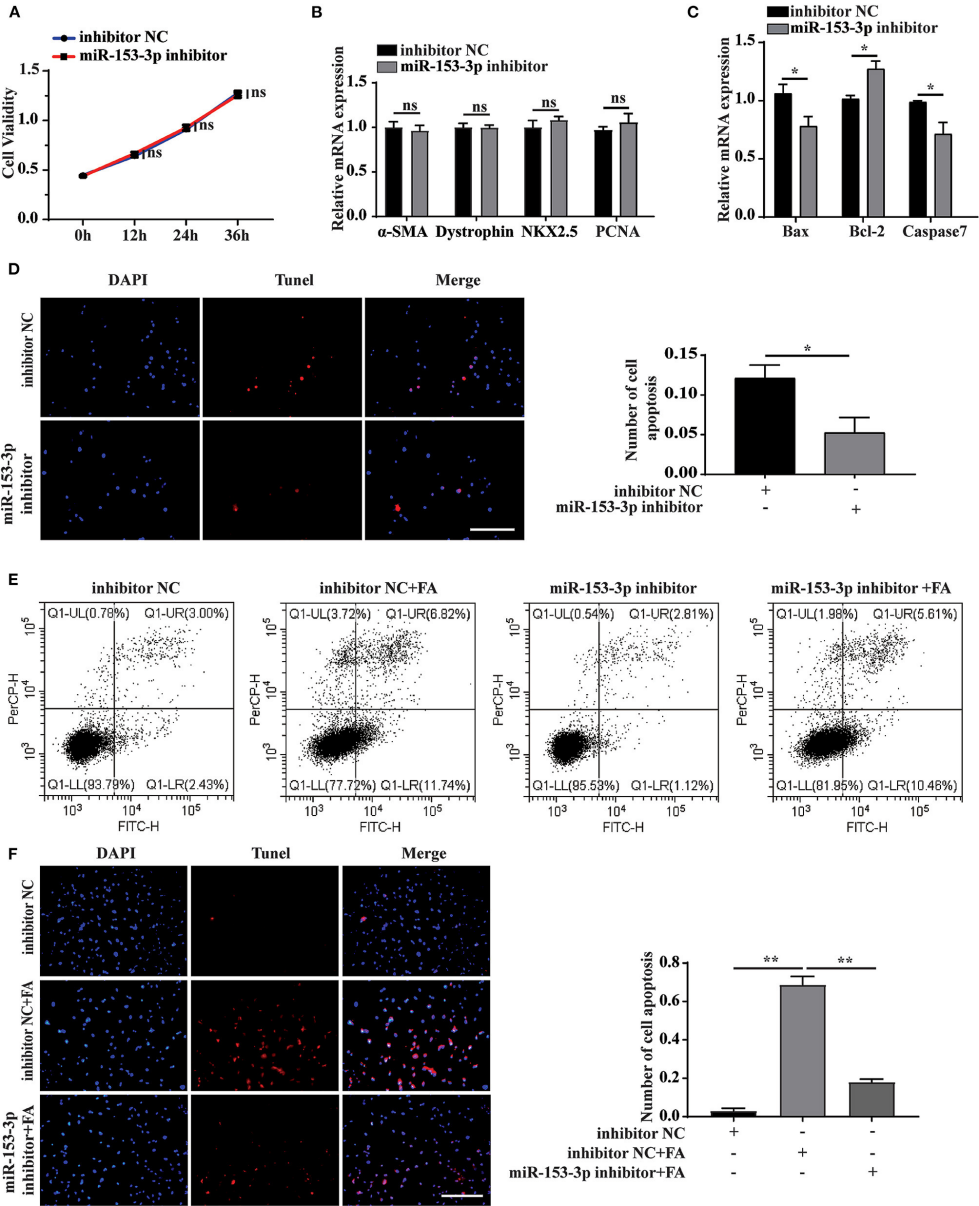
Knockdown of miR-153-3p inhibits apoptosis of H9C2 cells. **(A)** CCK8 detects the proliferation of H9C2 cells 0, 12, 24, and 36 h after miR-153-3p inhibitor transfection. **(B)** RT-qPCR was used to detect myocardial differentiation and the expression of proliferation markers α-SMA, Dystrophin, NKX2.5 and PCNA after miR-153-3p inhibitor transfected into H9C2 cells. **(C)** RT-qPCR to detect the expression of Bax, Bcl-2 and Caspase7 after miR-153-3p inhibitor transfection. **(D)** Tunel experiment to detect apoptosis after transfection of miR-153-3p inhibitor. **(E)** Cell flow cytometry was used to detect apoptosis after transfection of miR-153-3p inhibitor and continued to stimulate cell apoptosis with 150 μmol/L formaldehyde. **(F)** In Tunel experiment, after transfection of miR-153-3p inhibitor, 150 μmol/L formaldehyde stimulated cell apoptosis. Results were quantified by ImageJ software. Data are presented as mean ± SD. Scale bars, 200 μm. *n* = 3. *p < 0.05 vs. Ctl, **p < 0.01 vs. Ctl; ***p < 0.001 vs. Ctl.

We further explored the effect of miR-153-3p knockdown on cardiomyocyte function. Assessing cell proliferation at 0, 12, 24, and 36 h after transfecting H9C2 cells with the miR-153-3p inhibitor, the CCK-8 assay results indicated that cell proliferation was not significantly affected by transfection with the miR-153-3p inhibitor (Figure 4A). Consistently, transfecting H9C2 cells with the miR-153-3p inhibitor for 24 h did not significantly change the expression of the differentiation and proliferation markers, dystrophin, NKX2.5, and PCNA (Figure 4B). However, the expression of Bax and Caspase7 decreased, while that of Bcl-2 increased (Figure 4C). To further verify whether the miR-153-3p inhibitor inhibited apoptosis, TUNEL staining was employed to evaluate H9C2 cells transfected with the miR-153-3p inhibitor for 24 h. Compared with the control group, the miR-153-3p inhibitor-transfected group displayed fewer apoptotic cells (Figure 4D). Flow cytometry analysis of cells transfected with the miR-153-3p inhibitor for 24 h, followed by stimulation with 150 μmol/L FA for 24 h, revealed that transfection with the miR-153-3p inhibitor reversed FA-induced apoptosis (Figure 4E). These results were further confirmed by TUNEL staining (Figure 4F). Taken together, the results demonstrate that miR-153-3p knockdown inhibited the apoptosis of H9C2 cells.

### miR-153-3p Can Target βII Spectrin to Regulate Cardiomyocyte Apoptosis

**Figure 5:**
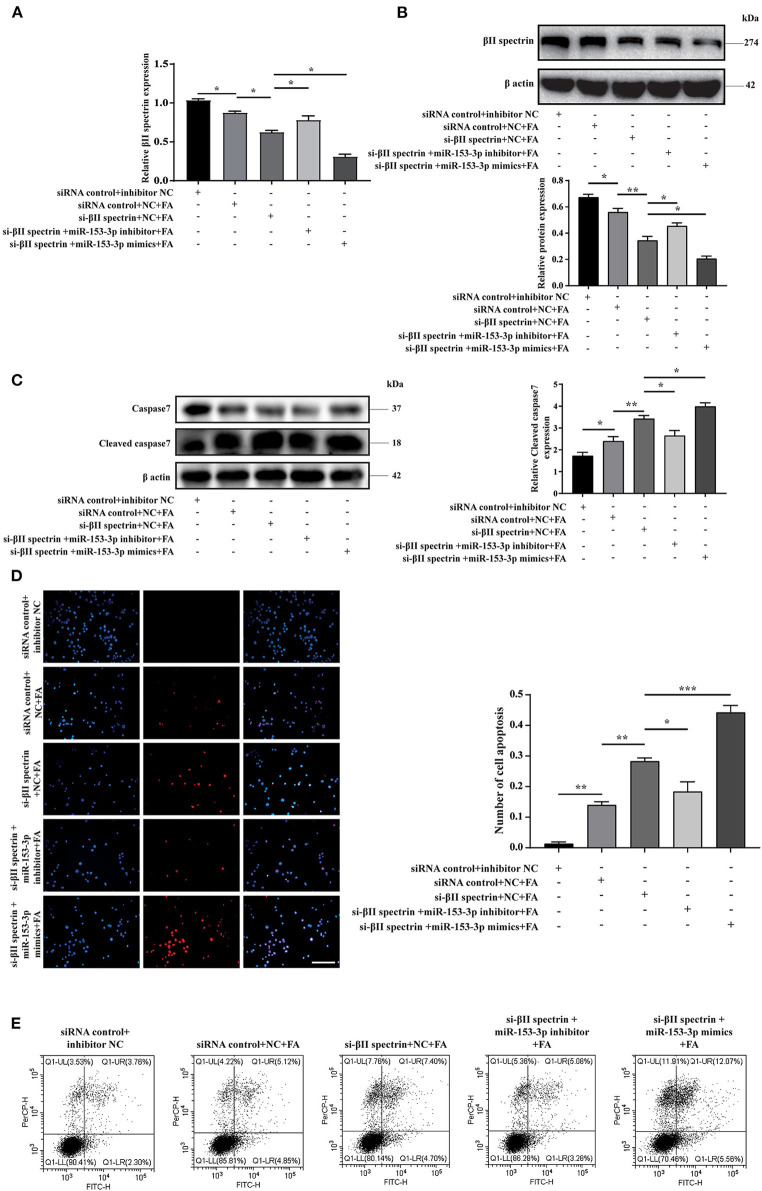
miR-153-3p can target βII spectrin to regulate cardiomyocyte apoptosis. **(A)** RT-qPCR was used to detect the expression of βII spectrin after co-transfection of Si-βII spectrin, miR-153-3p mimic or inhibitor, and treatment with formaldehyde (24 h). **(B)** WB detects the expression of βII spectrin after co-transfection of Si-βII spectrin, miR-153-3p mimic or inhibitor, and treatment with formaldehyde (24 h). **(C)** WB detects the expression of cleaved caspase7 after co-transfection of Si-βII spectrin, miR-153-3p mimic or inhibitor, and treatment with formaldehyde (24 h). **(D)** Tunel detects cell apoptosis after co-transfection of Si-βII spectrin, miR-153-3p mimic or inhibitor, and treatment with formaldehyde (24 h). **(E)** Cell flow cytometry was used to detect cell apoptosis after co-transfection of Si-βII spectrin, miR-153-3p mimic or inhibitor, and treated with formaldehyde (24 h). Results were quantified by ImageJ software. Data are presented as mean ± SD. Scale bars, 200 μm. *n* = 3. *p < 0.05 vs. Ctl, **p < 0.01 vs. Ctl; ***p < 0.001 vs. Ctl.

We further verified βII spectrin targeting of miR-153-3p under pathological stimuli. Cells were co-transfected with the βII spectrin inhibitor and the miR-153-3p mimic or inhibitor for 24 h and then were stimulated with FA for 24 h. We co-transfected H9C2 cells with the βII spectrin inhibitor designed by GenePharma and the miR-153-3p mimic or inhibitor, employing RT-qPCR and western blotting to detect changes in the expression of βII spectrin. βII spectrin expression was significantly reduced when cells were co-transfected with the miR-153-3p mimic and the βII spectrin inhibitor, and treated by FA (Figures 5A,B). WB showed that cleaved caspase7 expression was significantly increased in the group treated with βII spectrin inhibitor, miR-153-3p mimic, and FA, and transfection with miR-153-3p inhibitor reversed this phenomenon (Figure 5C). TUNEL staining indicated that the highest level of apoptosis was observed in the group treated with the βII spectrin inhibitor, miR-153-3p mimic, and FA, while transfection with the miR-153-3p inhibitor reversed the apoptosis (Figure 5D). Cell flow cytometry experiments further verified the above results (Figure 5E). The above confirmed that miR-153-3p can target βII spectrin to regulate cardiomyocyte apoptosis.

### Targeted Knockdown of miR-153-3p Inhibits the Occurrence of Formaldehyde-Induced Congenital Heart Disease in the Animal Model

**Figure 6:**
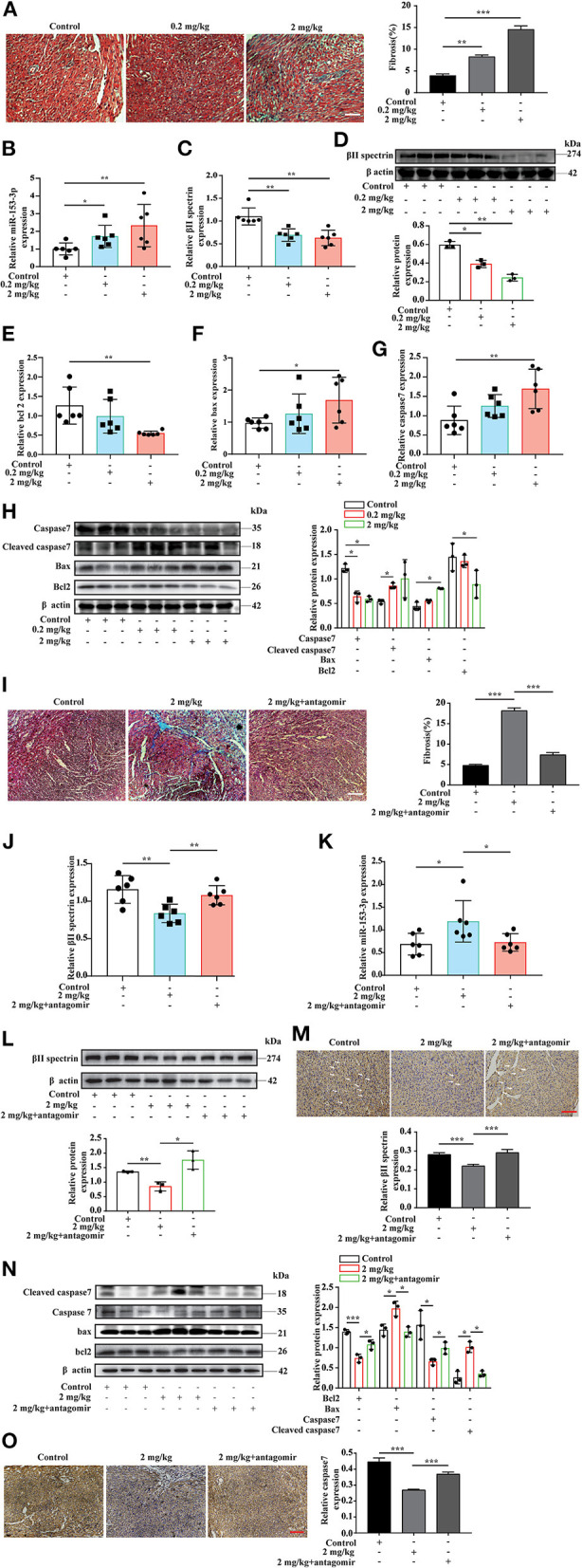
Targeted knockdown of miR-153-3p inhibits the occurrence of formaldehyde-induced congenital heart disease in the animal model. **(A)** Masson trichrome staining to detect myocardial tissue fibrosis (*n* = 3). **(B)** RT-qPCR detected increased expression of miR-153-3p in the myocardial tissue of fetal rats in the formaldehyde-treated group in animal experiments (*n* = 6). **(C)** RT-qPCR detected the decreased expression of βII spectrin in the myocardial tissue of fetal rats in the formaldehyde-treated group in animal experiments (*n* = 6). **(D)** The expression of βII spectrin in the myocardium of fetal rats in the formaldehyde-treated group was reduced by WB detection (*n* = 3). **(E)** RT-qPCR was used to detect the expression of Bcl 2 in the myocardial tissue of fetal rats in the formaldehyde-treated group (*n* = 6). **(F)** RT-qPCR was used to detect the expression of Bax in the myocardial tissue of fetal rats in the formaldehyde-treated group in animal experiments (*n* = 6). **(G)** RT-qPCR was used to detect the expression of Caspase7 in the myocardial tissue of fetal rats in the formaldehyde-treated group (*n* = 6). **(H)** WB was used to detect the expression of Caspase7, Cleaved caspase7, Bax and Bcl 2 in the myocardial tissue of fetal rats in the formaldehyde-treated group in animal experiments (*n* = 3). **(I)** Masson trichrome staining to detect myocardial tissue fibrosis (*n* = 3). **(J,K)** RT-qPCR detected the expression of βII spectrin and miR-153-3p in the myocardial tissue of fetal rats in medium concentration and miR-153-3p antagomir group in animal experiments (*n* = 6). **(L)** The expression of βII spectrin in the myocardium of fetal rats in recovery experiment by WB detection (*n* = 3). **(M)** Immunohistochemistry was used to detect the expression of βII spectrin in the myocardial tissue of fetal rats in recovery experiment (*n* = 3). **(N)** WB was used to detect the expression of Caspase7, Cleaved caspase7, Bax and Bcl 2 in the myocardial tissue of fetal rats in recovery experiment (*n* = 3). **(O)** Immunohistochemistry was used to detect the expression of caspase7 in the myocardial tissue of fetal rats in recovery experiment (n=3). Results were quantified by ImageJ software. Data are presented as mean ± SD. Scale bars, 200 μm. *n* = 3. *p < 0.05 vs. Ctl, **p < 0.01 vs. Ctl; ***p < 0.001 vs. Ctl.

The *in vitro* experiments investigated the interaction between miR-153-3p and βII spectrin when H9C2 cells were stimulated with FA. To further confirm these results in the animal model, we chose three FA concentrations for intraperitoneal injection based on previous research (29). During the experiment, the high FA concentration (20 mg/kg) resulted in a high fatality rate for pregnant rats and a low pregnancy rate; therefore, the low (0.2 mg/kg) and medium (2 mg/kg) FA concentration groups were selected for further analysis. Masson's trichrome staining revealed increased myocardial fibrosis in the FA treatment groups compared with the control group (Figure 6A). The RT-qPCR results indicated that βII spectrin expression decreased, while that of miR-153-3p increased in the FA treatment groups (Figures 6B,C). Western blotting revealed that βII spectrin expression decreased in the FA treatment groups (6D). In addition, Bax and caspase7 expression in the FA treatment group increased at the mRNA level, while that of Bcl-2 decreased, with significant changes observed in the group treated with 2 mg/kg FA (Figures 6E–G). At the protein level, the expression of Bcl-2 and caspase7 in the FA treatment group decreased, while that of cleaved caspase7 and Bax increased (Figure 6H). The *in vivo* results were consistent with those of the *in vitro* experiments, confirming that a certain concentration of FA promoted the occurrence of cardiomyocyte apoptosis.

To further verify the above results, we conducted an *in vivo* recovery experiment. The pregnant rats were divided into three treatment groups: control, medium FA concentration, and medium FA concentration plus miR-153-3p antagomir. As before, we used masson's trichrome staining revealed that compared with the FA treatment group, FA treatment and miR-153-3p antagomir group reversed myocardial fibrosis (Figure 6I). we used RT-qPCR to measure mRNA levels of βII spectrin and miR-153-3p expression in fetal heart tissues (Figures 6J,K). Western blotting indicated that βII spectrin expression in the medium FA concentration plus miR-153-3p antagomir group was recovered compared with that in the medium FA concentration group (Figure 6L). Immunohistochemical experiments also revealed that βII spectrin expression was restored (Figure 6M). Moreover, western blotting revealed that the expression of Bcl-2 and caspase7 in the medium FA concentration plus miR-153-3p antagomir group was recovered, while that of cleaved caspase7 and Bax decreased (Figure 6N). Immunohistochemical experiments also revealed that caspase7 expression was restored (Figure 6O). Altogether, the results indicated that miR-153-3p can directly target βII spectrin to regulate FA-induced cardiomyocyte apoptosis.

## Discussion

FA is ubiquitous in the environment, and exposure to high levels of FA during pregnancy can cause miscarriage and CHD. However, the mechanism by which FA induces CHD remains unclear; thus, studying the regulatory targets and signaling pathways of CHD is of great significance for the development of potential treatments.

The cardiac cytoskeleton is critical in maintaining the integrity, structure, and function of the myocardium under physiological and pathological conditions. βII spectrin, a key cell membrane skeletal protein, is essential for membrane integrity (17). βII spectrin deficiency is associated with severe heart disease, such as congenital arrhythmia, acquired and congenital heart failure, and possible sudden cardiac death (37). Emerging data indicate that βII spectrin is essential in embryonic heart development. The complete deletion of βII spectrin can lead to the death of mouse embryos, accompanied by a variety of defects, including abnormal development of liver, nerve, gastrointestinal tract, and angiogenesis (17). Heart size also reportedly differed significantly between wild-type and homozygous mutant embryos at embryonic day (E) 15.5, and histological studies revealed a thickened ventricular wall and failed blood vessel formation in the homozygous mutation group (32). Moreover, βII spectrin knockdown in homozygous mutant embryos interfered with heart cell differentiation and induced extensive apoptosis at E16.5 (32). In the current study, FA treatment significantly decreased βII spectrin expression in cardiomyocytes *in vivo* and *in vitro*, and significantly increased fetal rat heart fibrosis. Therefore, the above results strongly indicate that βII spectrin is essential for normal heart development and may provide a potential target for regulating CHD.

Recent studies have reported that apoptosis is closely associated with CHD. Apoptosis, which mediates the morphogenesis of tissues and organs in the human body, occurs in ventricular myocardium, cardiac nerves, or fibroblasts. Physiological apoptosis is necessary, but if apoptosis occurs excessively, the result is harmful. Little is known about signal regulation related to cardiac cell apoptosis; however, there is clear evidence that focal apoptosis is responsible for the development of the embryonic outflow tract, heart valve, conduction system, and coronary vasculature (38). Nox2 deficiency can lead to decreased levels of reactive oxygen species in the E10.5 heart and increased apoptosis (39). During embryogenesis, apoptosis is one of the key cellular events that regulates heart development. Decreased cardiomyocyte abundance and increased numbers of apoptotic cells under hyperglycemic conditions can cause heart defects (40). For example, maternal type 2 diabetes triggers excessive apoptosis in the ventricular myocardium, endocardial cushion, and embryonic heart outflow tract (41). Increasing evidence supports that apoptosis and cardiomyocyte remodeling are the main pathologies of CHD. In our study, βII spectrin participated in FA-induced cardiomyocyte apoptosis. Treatment with FA significantly reduced the number of H9C2 cells, while significantly increasing fetal heart fibrosis. Our results further indicate that apoptosis is critical in the development of the heart.

A large number of studies have demonstrated that miRNA is vital in heart development, but only a few have suggested that miRNA is involved in its pathogenesis. Injecting miRNA *in vivo* and *in vitro* can induce cardiac malformations and dysfunction, inhibit the growth of myocardial cells, and interfere with the normal development of the heart (42–44). In this study, FA exposure has no significant effect on the expression of PCNA. FA treatment significantly induced apoptosis of H9C2 cells. Through bioinformatics analysis and further experimental verification, we determined that FA significantly decreased βII spectrin expression, while significantly increasing miR-153-3p expression. Overexpression of miR-153-3p and knockdown of miR-153-3p have no significant effect on cardiomyocyte differentiation markers (α-SMA, dystrophin, and NKX2.5) and PCNA. Investigating the interaction and function of miR-153-3p and βII spectrin, and the caspase7/cleaved caspase7 and Bax/Bcl-2 apoptosis pathways, we determined for the first time that miR-153-3p targeting βII spectrin promoted the apoptosis of H9C2 cells. The expression of cleaved caspase7 and pro-apoptotic protein Bax increased, while the expression of anti-apoptotic protein Bcl-2 decreased. Therefore, the above findings support the potential involvement of miRNA in the development of the heart. Using an animal model, we investigated the effects of FA-induced cardiomyocyte apoptosis during embryonic development. Consistently, we found that miR-153-3p expression was significantly increased in fetal rat myocardial tissues after FA treatment, while βII spectrin expression was significantly decreased. The protein levels of Bax and cleaved caspase7 increased, while those of Bcl-2 and caspase7 decreased. In addition, intraperitoneal injection of FA during pregnancy significantly increased cardiac fibrosis in fetal rats. These findings further confirmed that FA exposure induced cardiomyocyte apoptosis during heart development. In addition, our recovery experiments revealed that injection of miR-153-3p antagomir inhibited the occurrence of FA-induced apoptosis during heart development and reduced the development of fetal heart fibrosis. Pharmacological intervention using miRNA oligonucleotides has been shown to improve cardiac contractility and reduce fibrosis, reducing cardiac dysfunction in patients with heart failure (44, 45). Additionally, miR-153-3p reportedly inhibits the translation of Mfn1, thereby accelerating mitochondrial fission and cardiomyocyte hypertrophy (46). These findings increase the possibility that miR-153-3p may provide a novel target for improving fibrosis-related cardiac dysfunction, especially CHD and cardiac hypertrophy.

More studies have shown the regulatory role of ncRNA in health and disease (47, 48). miRNA is a key regulator of cardiac phenotype that has caught the attention of basic scientists and clinicians (49–51). Technological advancements and a deeper understanding of the mechanisms regulating miRNA will enable crucial interpretation of miRNA functions, thus promoting their use for disease treatment (51–54). However, the molecular mechanisms and regulatory pathways associated with cardiomyocyte apoptosis and CHD require more research. Although the therapeutic potential of ncRNA has been noted (55–58), challenges remain before ncRNA can be applied as a target of CHD in clinical practice, such as changes in the length and mode of action of ncRNA, as well as the complex molecular mechanism of CHD. The delivery of ncRNA (59, 60), off-target effects, and RNA instability make a clinical application more difficult. The long-term and adverse effects of ncRNA therapy also warrant further investigation. Furthermore, the lack of sequence conservation among different species makes preclinical animal research more challenging. Based on the current difficulties, ncRNA is best suited as a potential marker of disease. Indeed, some ncRNA markers have been associated with various causes of cardiovascular disease (49, 61–64).

In conclusion, this study found for the first time that βII spectrin plays a regulatory role in FA-induced cardiomyocyte apoptosis, and revealed a new regulatory pathway in which miR-153-3p targets βII spectrin to negatively impact myocardium development. *In vitro* and *in vivo*, the expression of Bax and cleaved caspase7 increased, while that of caspase7 and Bcl-2 decreased during FA-induced cardiomyocyte apoptosis. Our results also demonstrated that FA promotes fibrosis of myocardial tissues during heart development. These findings provide new insights into the complex molecular mechanism of cardiomyocyte apoptosis in CHD.

## Data Availability Statement

The original contributions presented in the study are included in the article/supplementary material, further inquiries can be directed to the corresponding author/s.

## Ethics Statement

The animal study was reviewed and approved by the Affiliated Hospital of Qingdao University.

## Author Contributions

TY, ZJ, and YY conceptualized and designed the study. PY, XH, XS, YT, TZ, JM, XC, and QL performed experiments and collected data. PY, YY, and TY analyzed data, drafted, and edited manuscript. TY, ZJ, PY, PS, and YY reviewed and commented on the manuscript. All authors reviewed the manuscript and agreed the paper to be submitted.

## Funding

This work was supported by the National Natural Science Foundation of China (grant no. 81870331), the Natural Science Foundation of Shandong Province (grant no. ZR201911110516), and the Qingdao municipal science and technology bureau project (grant no. 21-1-4-rkjk-12-nsh, 19-6-1-2-nsh).

## Conflict of Interest

The authors declare that the research was conducted in the absence of any commercial or financial relationships that could be construed as a potential conflict of interest.

## Publisher's Note

All claims expressed in this article are solely those of the authors and do not necessarily represent those of their affiliated organizations, or those of the publisher, the editors and the reviewers. Any product that may be evaluated in this article, or claim that may be made by its manufacturer, is not guaranteed or endorsed by the publisher.
